# The Value of Residual Volume/Total Lung Capacity as an Indicator for Predicting Postoperative Lung Function in Non-Small Lung Cancer

**DOI:** 10.3390/jcm10184159

**Published:** 2021-09-15

**Authors:** Oh-Beom Kwon, Chang-Dong Yeo, Hwa-Young Lee, Hye-Seon Kang, Sung-Kyoung Kim, Ju-Sang Kim, Chan-Kwon Park, Sang-Haak Lee, Seung-Joon Kim, Jin-Woo Kim

**Affiliations:** 1 Division of Pulmonary, Critical Care and Sleep Medicine, Department of Internal Medicine, College of Medicine, The Catholic University of Korea, Seoul 06591, Korea; obkwon@catholic.ac.kr (O.-B.K.); brainyeo@catholic.ac.kr (C.-D.Y.); beyer_kr@catholic.ac.kr (H.-S.K.); kimskmd@gmail.com (S.-K.K.); kimjusang@catholic.ac.kr (J.-S.K.); ckpaul@catholic.ac.kr (C.-K.P.); agmante@gmail.com (S.-H.L.); cmcksj@catholic.ac.kr (S.-J.K.); 2 Division of Allergy, Department of Internal Medicine, College of Medicine, The Catholic University of Korea, Seoul 06591, Korea; lehwyo@catholic.ac.kr; 3 Cancer Research Institute, College of Medicine, The Catholic University of Korea, Seoul 06591, Korea; 4 Division of Pulmonology, Department of Internal Medicine, Seoul St. Mary’s Hospital, Seoul 06591, Korea; 5 Postech-Catholic Biomedical Engineering Institute, Songeui Multiplex Hall, College of Medicine, The Catholic University of Korea, Seoul 06591, Korea

**Keywords:** lung cancer, COPD, postoperative lung function, hyperinflation

## Abstract

Chronic obstructive pulmonary disease (COPD) is one of the most frequently occurring concomitant diseases in patients with non-small cell lung cancer (NSCLC). It is characterized by small airways and the hyperinflation of the lung. Patients with hyperinflated lung tend to have more reserved lung function than conventionally predicted after lung cancer surgery. The aim of this study was to identify other indicators in predicting postoperative lung function after lung resection for lung cancer. Patients with NSCLC who underwent curative lobectomy with mediastinal lymph node dissection from 2017 to 2019 were included. Predicted postoperative FEV_1_ (ppoFEV_1_) was calculated using the formula: preoperative FEV_1_ × (19 segments-the number of segments to be removed) ÷ 19. The difference between the measured postoperative FEV_1_ and ppoFEV_1_ was defined as an outcome. Patients were categorized into two groups: preserved FEV_1_ if the difference was positive and non-preserved FEV_1_, if otherwise. In total, 238 patients were included: 74 (31.1%) in the FEV_1_ non-preserved group and 164 (68.9%) in the FEV_1_ preserved group. The proportion of preoperative residual volume (RV)/total lung capacity (TLC) ≥ 40% in the FEV_1_ non-preserved group (21.4%) was lower than in the preserved group (36.1%) (*p* = 0.03). In logistic regression analysis, preoperative RV/TLC ≥ 40% was related to postoperative FEV1 preservation. (adjusted OR, 2.02, *p* = 0.041). Linear regression analysis suggested that preoperative RV/TLC was positively correlated with a significant difference. (*p* = 0.004) Preoperative RV/TLC ≥ 40% was an independent predictor of preserved lung function in patients undergoing curative lobectomy with mediastinal lymph node dissection. Preoperative RV/TLC is positively correlated with postoperative lung function.

## 1. Introduction

Lung cancer can be classified into two major classes: non-small cell lung cancer (NSCLC) and small cell lung cancer (SCLC) [1]. Surgical resection is the standard treatment for early stage NSCLC [2]. Chronic obstructive pulmonary disease (COPD) is an important risk factor for lung cancer and is one of the most frequently occurring concomitant diseases in patients with lung cancer [3,4]. COPD is characterized by chronic airflow limitation due to small airways (obstructive bronchiolitis) and parenchymal destruction (emphysema) [5]. Lung volume reduction surgery (LVRS) is an accepted therapeutic option for patients with severe emphysema to relieve symptoms such as dyspnea and increased work of breathing. Spiration valve system (SVS) is a bronchoscopic lung volume reduction therapy, and it results in significant improvements in postoperative forced expiratory volume in one second (FEV_1_) and symptoms [6,7,8]. In order to predict postoperative lung function, the results of these studies indicating that reducing lung volume improves postoperative lung function should be considered.

Patients with COPD are known to manifest worse survival outcomes than patients without COPD in NSCLC treated with surgical resection [9]. Nevertheless, with the exception of surgical resection, no other treatment options are available to most of the patients with early stage NSCLC with COPD [10]. Since postoperative lung function is related to postoperative complications, postoperative changes in quality of life (QOL), and perioperative mortality, predicting postoperative lung function is crucial, and the treatment strategy should be established accordingly.

Predicted postoperative FEV_1_ (ppoFEV_1_) was used to predict postoperative pulmonary function for the assessment of perioperative risk. The ppoFEV_1_ can be calculated using a formula based on the number of resected segments [11,12]. However, COPD patients who underwent surgery had a relatively more preserved postoperative FEV_1_ than non-COPD patients did [13]. Previous studies reported that non-squamous cell histology, upper lobe resection, and current smoking status were related to poor postoperative outcomes [14,15,16,17]. These factors have insufficient accuracy in predicting postoperative lung function, and there is no accurate method available to predict postoperative lung function. This suggests the need for accurate clinical prognostic indicators. The objective of this study was to identify other prognostic indicators that can complement ppoFEV_1_ to increase the prediction accuracy of postoperative lung function.

## 2. Materials and Methods

### 2.1. Study Population

A total of 238 patients with lung cancer who underwent curative lobectomy with mediastinal lymph node dissection at seven hospitals in the Catholic University of Korea (Seoul St. Mary’s Hospital, Incheon St. Mary’s Hospital, Yeouido St. Mary’s Hospital, Eunpyeong St. Mary’s Hospital, Bucheon St. Mary’s Hospital, St. Vincent’s Hospital, and Uijeongbu St. Mary’s Hospital) from 2017 to 2019 were included. The inclusion criteria were pathologically confirmed primary NSCLC, no relapse up to 12 months after surgery, and R0 resection. Relapse was defined based on radiological or histological evidence of cancer within 12 months after treatment [18,19]. The study included 238 patients who underwent pulmonary function test (PFT) during the evaluation period (6 ± 3 months). Incomplete records of 62 patients were detected in the second evaluation period (12 ± 3 months). We analyzed the PFT data of 176 patients during the second evaluation period. This study was approved by the Institutional Review Board (IRB) of the Catholic University of Korea. (IRB: XC20RIDI0137). The requirement for informed consent was waived due to the retrospective nature of the study.

### 2.2. Data and Outcome Definition 

Demographic data including age and gender, Eastern Cooperative Oncology Group (ECOG) Performance Scale, histologic features, smoking history, post-operative tumor stage according to the eighth edition of the tumor–node–metastasis (TNM) classification [20], cancer location, and PFT results were collected. FEV_1_, FEV_1_/forced vital capacity (FVC), the diffusing capacity of the lung for carbon monoxide (DL_CO_), and the residual volume (RV)/total lung capacity (TLC) were measured three times: before surgery, 6 ± 3 months (abbreviated as 6 months for convenience) after surgery, and 12 ± 3 months (abbreviated as 12 months) after surgery. Based on smoking history, the patients were grouped into never smokers if they never smoked or if they had smoked less than 100 cigarettes in their lifetime, and ever smokers if they had smoked at least 100 cigarettes in their lifetime [18,21]. PFT was performed in accordance with the American Thoracic Society/European Respiratory Society standardization guidelines. In order to compare the postoperative lung function with ppoFEV_1,_ ppoFEV_1_ was calculated using the formula: preoperative FEV_1_ × (19 segments-the number of segments to be removed) ÷ 19. The number of segments for each lobe was: right upper lobe, three; right middle lobe, two; right lower lobe, five; left upper lobe, five; and left lower lobe, four [11,12]. Difference was defined as the postoperative FEV_1_ minus ppoFEV_1_. Outcome was defined as preserved FEV_1_ if the difference was positive and non-preserved FEV_1_ if the difference was negative, and the patients were categorized accordingly.

### 2.3. Predictive Factors

If lung cancer was located in the right upper lung (RUL) or the left upper lung (LUL), it was categorized as both upper lungs (BUL). Preoperative FEV_1_ ≥ 80%, FEV_1_/FVC ≥ 70%, and DL_CO_ ≥ 80% were used as cut-off values to categorize preoperative lung function and to analyze factors associated with postoperative lung function. Preoperative RV/TLC ≥ 40% was used to represent pulmonary hyperinflation [22].

### 2.4. Statistical Analysis

All statistical analyses were performed using R software, version 4.0.5. (R Foundation for Statistical Computing, Vienna, Austria) Continuous variables are presented as means with standard deviation. Categorical variables are expressed as numbers with percentages. Patients were categorized into two groups: FEV_1_ preserved and FEV_1_ non-preserved. The unpaired Student’s *t* test was used for the comparison of continuous variables between the two groups. The Chi-squared test was performed for the analysis of categorical variables. Univariate and multivariate logistic regression analyses were used to determine the factors associated with preserved FEV_1_ at 6 months after surgery. All variables with *p* < 0.2 in the univariate analysis were included in the multivariate analysis. The odds ratio (OR) and a 95% confidence interval (CI) were computed for each category. Univariate linear regression was performed to assess the individual effects of the pulmonary lung function variables and hyperinflation on FEV_1_ after surgery. All of the variables were entered into multiple linear regression analysis. P values less than 0.05 were considered statistically significant for all analyses.

## 3. Results

### 3.1. Overall Patient Characteristics

A total of 238 patients with primary lung cancer who received a curative lobectomy with mediastinal lymph node dissection and did not relapse up to 12 months after surgery were included. The patient characteristics are presented in Table 1. The mean age of the patients was 66.7 years, and the majority of the patients (60.5%) were male. Most of the patients had an ECOG performance scale of 0–1 (99.2%), and adenocarcinoma was the dominant histologic feature (69.7%). In regard to smoking status, the proportions of patients who were never smokers and ever smokers were 39.5% and 60.5%, respectively. Stage I was the most common stage (66.8%), and the LUL was the most common lung cancer location (29.4%). The second most common lung cancer site was the RUL (26.9%). The preoperative PFT results were: FEV_1_ 97.8 ± 21.5 %; FEV_1_/FVC 73.2 ± 9.5 %; DL_CO_ 88.2 ± 19.1%; and RV/TLC 35.9 ± 8.7%.

### 3.2. Comparison of FEV_1_ Non-Preserved and Preserved Groups

A comparison of the two groups presented in Table 2 reveals that 74 patients (31.1%) had non-preserved FEV_1_, while 164 (68.9%) had preserved FEV_1_. The mean age (*p* = 0.44), sex distribution (*p* = 0.73), ECOG performance scale (*p* = 0.56), histologic features (*p* = 0.09), smoking status (*p* = 0.52), stage (*p* = 0.64), and lung cancer location (*p* = 0.16) did not differ between the two groups. The proportion of preoperative RV/TLC ≥ 40% in the FEV_1_ non-preserved group (21.4%) was lower than in the preserved group (36.1%) (*p* = 0.03).

### 3.3. Factors Associated with Preserved Postoperative FEV_1_

The results of the logistic regression analysis identifying the factors associated with postoperative FEV_1_ preservation are presented in Table 3. Histologic features (unadjusted OR 1.84, 95% CI 0.90–3.73, *p* = 0.093), location of lung cancer (unadjusted OR 0.67, 95% CI 0.38–1.17, *p* = 0.157), and preoperative RV/TLC ≥ 40% (unadjusted OR 2.11, 95% CI 1.09–4.06, *p* = 0.026) had a *p* value less than 0.2 in the univariate analysis. Only preoperative RV//TLC ≥ 40% (adjusted OR 2.02, 95% CI 1.03–3.97, *p* = 0.041) remained statistically significant in multivariate analysis.

### 3.4. Correlation of Differences with Preoperative PFT Parameters and Postoperative FEV_1_

**Figure 1:**
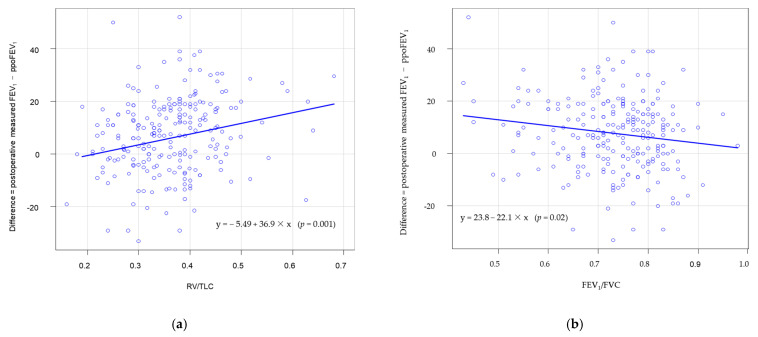
Correlation of difference with (**a**) RV/TLC and (**b**) FEV_1_/FVC.

In the univariate linear regression analysis (Table 4), preoperative FEV_1_ (*p* = 0.032), FEV_1_/FVC (*p* = 0.020) showed a significant negative correlation, and RV/TLC (*p* = 0.001) showed a significant positive correlation with a difference. The scatter plot for difference and RV/TLC is presented in Figure 1a. The scatter plot for FEV_1_/FVC is provided in Figure 1b. In the multiple linear regression analysis (Table 4), RV/TLC was the only significant variable that was positively correlated with statistically significant difference (*p* = 0.004).

## 4. Discussion

In this study, we found that RV/TLC was a prognostic indicator for predicting postoperative lung function by comparing the postoperative measured FEV_1_ with the conventional method of prediction and that it can be calculated using a simple formula [11]. Further, only the correlation between RV/TLC and postoperative lung function showed statistical significance, while the correlation with other conventional variables such as preoperative FEV_1_, FEV_1_/FVC, and DL_CO_ were not statistically significant. These results suggest that traditional prediction methods are insufficient and that hyperinflation, which can be assessed by RV/TLC, is an important factor to predict postoperative lung function. Since RV/TLC is correlated with postoperative lung functions, it can be treated as an treatment plan indicator.

Preoperative RV/TLC ≥ 40% is associated with more frequent exacerbations and is an independent risk factor for all-cause mortality in COPD [23]. In order to compare the extent of postoperative FEV_1_ changes in a hyperinflated lung with that of the normal lung, RV/TLC ≥ 40% was considered as a prognostic indicator. Patients with preoperative RV/TLC ≥ 40% had reserved lung function that was greater than predicted at 6 months after surgery. Patients with emphysema manifest low preoperative FEV_1_ and might be considered surgically contraindicated. However, previous studies have shown that surgery can improve postoperative lung function [24]. Surgery reduced hyperinflation, which increased the elastic recoil and global inspiratory muscle strength, which resulted in symptom improvement and reserved lung function [6].

In our study, a total of 55.3% of the patient population had lung cancer involving the upper lobe (29.4% in LUL, 26.9% in RUL). Previous studies have shown that volume loss was larger in upper lobectomy than in lower lobectomy, and lower lobectomy led to better postoperative compensation [16]. Upper lobectomy leads to an upward displacement of the diaphragm and the remaining lung, resulting in bronchial kinking and obstruction and worse postoperative lung function [25,26]. When cancer was located at either of the upper lobes, the OR was 0.67 in the univariate analysis and was 0.68 for the multivariate analysis, which was consistent with previous studies. However, the sample size was not large enough to reach statistical significance (Table 3).

Surgery is considered as the first choice of treatment for lung cancer and is associated with the lowest mortality rate compared with other modalities [2,27]. However, patients with comorbidities tend to manifest higher postoperative morbidity and mortality after surgery [28,29]. The prevalence of COPD in patients with lung cancer was 40–70%. Due to the increased prevalence of COPD and lung cancer, the number of surgeries involving patients with reduced lung function also increased [3,4,27]. Surgical resection in these patients with lower FEV_1_ might lead to respiratory failure and therefore requires careful evaluation of postoperative lung function. Since postoperative function is predicted by the proportion of resected lobes, a low preoperative FEV_1_ might lead to limited resection, which can result in poor survival rates [14].

However, resection surgery can improve lung function in patients with COPD by reducing pulmonary hyperinflation [6,7,14]. To evaluate lung function, FEV_1_/FVC is used to measure airway obstruction, and RV/TLC is used to measure lung hyperinflation [22]. COPD is an obstructive lung disease that results in low FEV_1_/FVC, whereas emphysematous lung is hyperinflated, which results in high RV/TLC. FEV_1_/FVC is usually used to estimate the severity of COPD because it represents the degree of airway obstruction. In this study, we measured preoperative RV/TLC ≥ 40% to represent hyperinflation [22]. Patients with preoperative RV/TLC ≥ 40% had reserved lung function compared to ppoFEV_1_, which implies that patients deemed inoperable with low preoperative FEV_1_ due to a hyperinflated lung might be considered operable. Therefore, RV/TLC calculation can increase the accuracy of predicting postoperative lung function.

The study has several limitations, but these were trivial due to the following reasons: It was a short-term study lasting up to 6 months after surgery, and the had a bias due to missing data. We also evaluated 12-month data, but they was not fully analyzed due to missing values (data not shown). In our study, postoperative FEV_1_ was compared with ppoFEV_1_, which was calculated using the formula. A ventilation–perfusion scan can predict postoperative FEV_1_ more accurately than the formula can [30]. However, ventilation–perfusion scan data were not available in this study. Future studies should compare postoperative measured FEV_1_ with ppoFEV_1_ using ventilation–perfusion scan. The method of surgery (open thoracotomy or video-assisted thoracoscopic surgery) was not distinguished. However, video-assisted thoracoscopic surgery was performed unless contraindications existed at the hospitals, as previously indicated. Patients received adjuvant therapies, including chemotherapy and radiotherapy, according to the National Comprehensive Cancer Network (NCCN) guidelines, but the type of adjuvant therapy was not considered as a factor in this study [31]. This factor can affect post-operative lung function and therefore is another limitation of this study, suggesting the need for further studies.

## 5. Conclusions

Preoperative RV/TLC ≥ 40% was an independent predictor of reserved lung function in patients undergoing curative lobectomy with mediastinal lymph node dissection, and it was positively correlated with postoperative FEV_1_. Further large-scale studies are required to predict postoperative lung function and the prognosis of patients after surgery.

## Figures and Tables

**Table 1:** Clinical characteristics of the study patients.

	Overall Patients (*n* = 238)
Age, years, mean ± SD	66.7 ± 8.9
Sex, *n* (%)	
Male	144 (60.5)
Female	94 (39.5)
ECOG performance status, *n* (%)	
0 and 1	236 (99.2)
≥2	2 (0.8)
Histologic features, *n* (%)	
Adenocarcinoma	166 (69.7)
Squamous cell carcinoma	55 (23.1)
Others	17 (7.1)
Smoking, *n* (%)	
Never smoker	94 (39.5)
Ever smoker	144 (60.5)
Stage, *n* (%)	
I	159 (66.8)
II	49 (20.6)
III	30 (12.6)
Location, *n* (%)	
RUL	64 (26.9)
RML	14 (5.9)
RLL	48 (20.2)
LUL	70 (29.4)
LLL	42(17.6)
PFT	
FEV_1_ (pre), %, mean ± SD	97.8 ± 21.5
FEV_1_/FVC, %, mean ± SD	73.2 ± 9.5
DLco, %, mean ± SD	88.2 ± 19.1
RV/TLC, %, mean ± SD	35.9 ± 8.7

SD, standard deviation; ECOG, Eastern Cooperative Oncology Group; RUL, right upper lobe; RML, right middle lobe; RLL, right lower lobe; LUL, left upper lobe; LLL, left lower lobe; PFT, pulmonary function test; FEV_1_, forced expiratory volume in one second; FVC, forced vital capacity; DLco, diffusing capacity of the lung for carbon monoxide; RV, residual volume; TLC, total lung capacity.

**Table 2:** Comparison between the FEV_1_ non-preserved and preserved groups.

	POD 6 Months (*n* = 238)		
	FEV_1_ Non-Preserved *n* = 74 (31.1%)	FEV_1_ Preserved *n* = 164 (68.9%)	*p*-Value
Age, years, mean ± SD	66.0 ± 8.6	67.0 ± 9.0	0.44
Sex, *n* (%)			0.73
Male	46 (61.1)	98 (59.8)	
Female	28 (37.8)	66 (40.2)	
ECOG performance status, *n* (%)			0.56
0 and 1	73 (98.6)	163 (99.4)	
≥2	1 (1.4)	1 (0.6)	
Histologic features, *n* (%)			0.09
Sqcc	12 (16.2)	43 (26.2)	
Non-Sqcc	62 (83.8)	121 (73.8)	
Smoking, *n* (%)			0.52
Never	27 (36.5)	67 (40.9)	
Ever	47 (63.5)	97 (59.1)	
Stage, *n* (%)			0.64
I	51 (68.9)	108 (65.9)	
II–III	23 (31.1)	56 (34.1)	
Location, *n* (%)			0.16
BUL	47 (63.5)	88 (53.7)	
Others	27 (36.5)	76(46.3)	
Preoperative PFT			
FEV_1_, %, mean ± SD	100.2 ± 19.8	96.7 ± 22.2	0.24
FEV_1_/FVC, %, mean ± SD	74.1 ± 8.9	72.8 ± 9.8	0.34
DLco, %, mean ± SD	89.1 ± 17.7	87.8 ± 20.9	0.64
RV/TLC ≥ 40%, *n* (%)	15 (21.4)	57 (36.1)	0.03

POD, post-operative days; SD, standard deviation; ECOG, Eastern Cooperative Oncology Group; Sqcc, squamous cell carcinoma; BUL, both upper lobe; PFT, pulmonary function test; FEV_1_, forced expiratory volume in one second; FVC, forced vital capacity; DLco, diffusing capacity of the lung for carbon monoxide; RV, residual volume; TLC, total lung capacity.

**Table 3:** Factors associated with preserved postoperative FEV_1_.

	POD 6 Months (*n* = 238)					
	Univariate Analysis			Multivariate Analysis		
	OR	95% CI	*p*-Value	OR	95% CI	*p*-Value
Age	1.01	0.98–1.04	0.434			
Sex (Female vs. Male)	1.11	0.63–1.94	0.725			
ECOG (≥2 vs. 0–1)	0.45	0.03–7.26	0.572			
Histology (Sqcc vs. Non-sqcc)	1.84	0.90–3.73	0.093	1.42	0.68–2.97	0.357
Smoking (Ever vs. Never)	0.83	0.47–1.46	0.524			
Stage (II–III vs. I)	1.15	0.64–2.07	0.642			
Location (BUL vs. other)	0.67	0.38–1.17	0.157	0.68	0.38–1.22	0.199
Preoperative FEV_1_ (<80% vs. ≥80%)	1.50	0.71–3.15	0.287			
Preoperative FEV_1_/FVC (<70% vs. ≥70%)	1.05	0.57–1.95	0.871			
Preoperative DLco (<80% vs. ≥80%)	1.19	0.66–2.16	0.565			
Preoperative RV/TLC (≥40% vs. <40%)	2.11	1.09–4.06	0.026	2.02	1.03–3.97	0.041

POD, post-operative days; ECOG, Eastern Cooperative Oncology Group; Sqcc, squamous cell carcinoma; Ever, current and former; BUL, both upper lobe; FEV_1_, forced expiratory volume in one second; FVC, forced vital capacity; DLco, diffusing capacity of the lung for carbon monoxide; RV, residual volume; TLC, total lung capacity.

**Table 4:** Correlation of differences (postoperative FEV_1_ minus ppoFEV_1_) with preoperative PFT parameters and postoperative FEV_1_.

Preoperative Variables	POD 6 Months				
	Univariate		Multivariate		
	Β ± SE	*p*-Value	Β ± SE	Partial R^2^	*p*-Value
FEV_1_	−0.091 ± 0.042	0.032	−0.001 ± 0.057	0.000	0.986
FEV_1_/FVC	−22.129 ± 9.463	0.020	−15.280 ± 11.310	0.008	0.178
DL_CO_	−0.050 ± 0.046	0.281	−0.033 ± 0.051	0.003	0.513
RV/TLC	36.895 ± 10.505	0.001	33.051 ± 11.388	0.048	0.004

## Data Availability

The data presented in this study are available on request from the corresponding author.
